# Molecular basis of human cerebral malaria development

**DOI:** 10.1186/s41182-016-0033-6

**Published:** 2016-09-27

**Authors:** Saw Thu Wah, Hathairad Hananantachai, Usanee Kerdpin, Chotiros Plabplueng, Virapong Prachayasittikul, Pornlada Nuchnoi

**Affiliations:** 1 Department of Clinical Microscopy, Faculty of Medical Technology, Mahidol University, Bangkok, 10700 Thailand; 2 Faculty of Tropical Medicine, Mahidol University, Bangkok, Thailand; 3 Department of Chemistry, Faculty of Science, Naresuan University, Phitsanulok, 65000 Thailand; 4 Center for Research and Innovation, Faculty of Medical Technology, Mahidol University, Bangkok, Thailand; 5 Department of Clinical Microbiology and Applied Technology, Faculty of Medical Technology, Mahidol University, Bangkok, Thailand

**Keywords:** Cerebral malaria, Genetics, Pathogenesis, MicroRNA

## Abstract

Cerebral malaria is still a deleterious health problem in tropical countries. The wide spread of malarial drug resistance and the lack of an effective vaccine are obstacles for disease management and prevention. Parasite and human genetic factors play important roles in malaria susceptibility and disease severity. The malaria parasite exerted a potent selective signature on the human genome, which is apparent in the genetic polymorphism landscape of genes related to pathogenesis. Currently, much genomic data and a novel body of knowledge, including the identification of microRNAs, are being increasingly accumulated for the development of laboratory testing cassettes for cerebral malaria prevention. Therefore, understanding of the underlying complex molecular basis of cerebral malaria is important for the design of strategy for cerebral malaria treatment and control.

## Background

Cerebral malaria (CM) is a life-threatening disease that represents a global health problem particularly in tropical countries. According to a report of the World Health Organization (WHO) in the year 2015, malaria transmission still occurs in approximately 97 countries and territories, mostly in Sub-Saharan Africa, Southeast Asia, and South America. In the year 2013, the estimated incidence of malaria infection was 198 million cases (range 124–283 million) worldwide. Over 575,000 cases of CM have been reported. Approximately 584,000 cases (range 367,000–755,000) died from malaria. African children are the most affected case of CM. Most of the malaria-related deaths, approximately 90 %, occurred in Africa [1]. In Sub-Saharan Africa, 575,000 children with CM have been annually reported and 110,000 cases died (approximately 19–25 % case fatality rate). Unfortunately, >2 % of survivors of CM experienced developmental and behavioral impairment lasting for 6 months. The disability, severity, and neurological duration are critical for CM management and essential for understanding of CM pathogenesis. Due to prevention and control programs, the morbidity and motility rates of malaria were reduced globally. In Thailand, the mortality rate decreased 50–74 % between 2000 and 2013 [1, 2].

*Plasmodium falciparum* is the causative organism leading to human CM development. The bite of a *P. falciparum*-infected female anopheline mosquito mediates the development of various disease severities ranging from uncomplicated malaria to severe malaria and CM. Uncomplicated malaria or mild malaria is defined as a febrile illness without any clinical or laboratory signs of severity or vital organ dysfunction. Complicated malaria or severe malaria involves the central nervous system (cerebral malaria), the pulmonary system (respiratory failure), the renal system (acute renal failure), and the hematopoietic system (severe anemia). According to the updated definition of severe falciparum malaria by the WHO (2015), severe falciparum malaria is defined as the presence of *P. falciparum* asexual parasitemia, with one or more clinical features or laboratory findings (Table 1) and without any identified alternative causes. The hallmarks of CM are coma (Glasgow Coma Scale <11, Blantyre coma score <3) or malaria with a persistent coma [3]. Clinical manifestations of severe malaria include but are not limited to CM (with incidence rate of 0.9–3.5 per 1000 child-year), severe malarial anemia (12–50 per 1000 child-year), and respiratory failure (1.4–5.4 per 1000 child-year) [1, 2].

**Table 1:** WHO guideline for complicated malaria

Clinical features
- Impaired consciousness: Glasgow Coma Scale <11 in adults or Blantyre coma score <3 in children - Prostration - Multiple convulsion: >2 episodes within 24 h - Respiratory distress - Pulmonary edema: radiologically confirmed or oxygen saturation <92 % in room air with a respiratory rate >30/min - Significant bleeding: including recurrent or prolonged bleeding from the nose, gums, and venipuncture sites; hematemesis or melena - Shock
Laboratory findings
- Metabolic acidosis: plasma bicarbonate <15 mmol/L or venous plasma lactate ≥5 mmol/L - Hypoglycemia: blood glucose <2.2 mmol/L or 40 mg/dL - Severe malarial anemia: Hb <5 g/dL, Hct ≤15 % in children (<12 years of age) with parasite >10,000/μL; Hb <7 g/dL, Hct ≤20 % in adults with parasite >10,000/μL - Renal impairment: plasma or serum creatinine >265 μmol/L (3 mg/dL) or blood urea >20 mmol/L - Jaundice: plasma or serum bilirubin >50 μmol/L (3 mg/dL) with parasite count >10,000/μL - Hyperparasitemia: *P. falciparum* parasitemia >10 %

## Mechanism of CM

The mechanism of CM is not clearly understood. Researchers have exerted extensive efforts to elucidate the mechanism of CM using several approaches such as in vivo experimental mouse models [4, 5], in vitro co-cultures of parasitized red cells with human brain microvascular endothelial cells [6, 7], and postmortem tissue and clinical samples from patients in endemic areas [8, 9]. For the in vivo studies, *Plasmodium berghei* ANKA (PbA)-infected CBA and C57BL/6 mice have been used as a CM susceptible model. The mouse model manifests the neurological symptoms within 6–14 days after infection and then dies [10]. The differences in the characteristics between the murine model and humans are the host receptor and the absence of knob-like structures of the *P. falciparum* erythrocyte membrane protein 1 (PfEMP1) (Table 2) [11].

**Table 2:** Characteristic of *P. berghei* ANKA strain in mouse model and *P. falciparum* in humans for CM study

Characteristics	*P. berghei* ANKA model	Human CM
Stage of sequestration	Schizonts	Mature trophozoites, schizonts, mature gametocytes
Host cell adhesion molecule	CD36	CD36, ICAM-1, PCAM-1/CD31, CR1, CSA
Parasite ligand	No homologous protein of PfEMP1	PfEMP1
Site for sequestration	Brain, lung, spleen, adipose tissue	Brain, lung, spleen, intestine, bone marrow, skin, skeletal and cardiac muscle, and adipose tissue
Brain hemorrhage	++	++
Obstruction of microvessels	+++	+++
Sequestration of infected RBC	+	+++
Knobs on infected RBC	−	++

−, +, ++, +++ indicate degree of characteristics

The development of CM is biologically complex and involves multiple mechanisms such as sequestration, immunopathology by the pro-inflammatory cytokine interferon-γ (IFN-γ), tumor necrosis factor alpha (TNF-α), and apoptosis. Red cell sequestration is an important step in the development of CM. The binding of PfEMP1 on infected red blood cells to host receptors such as intercellular adhesion molecule-1 (ICAM-1) and CD36 on brain endothelial cells mediates sequestration [12, 13]. Parasitized red blood cells (pRBCs) also form rosettes and clumps that impair microcirculation and cause hypoxia, which leads to neuronal tissue necrosis [14]. The production of IFN-γ from Th1 cells stimulates monocytes to express higher levels of the transmembrane form of TNF (memTNF) that interacts with tissue necrotic factor receptor 2 (TNFR2) expressed on brain endothelial cells causing the up-regulation of ICAM-1 on brain endothelial cells [15]. The up-regulation of ICAM-I on brain endothelial cells leads to an increase in platelets, pRBCs, and leukocyte adhesion. This contributes to the following clinical outcomes: (1) vessel obstruction and ischemia and (2) vessel disruption and brain hemorrhage. There are several reports of apoptotic mechanisms in endothelial cells leading to blood-brain barrier (BBB) dysfunctions in CM. The consequence of pRBC cytoadherence to the endothelial cell leads to (1) caspase 8 and 9 activation leading to apoptosis, (2) cytotoxic T cell activation leading to perforin-mediated cell death [16], (3) TNF-α overproduction as a result of the glycosylphosphatidylinositol (GPI activates apoptosis by inducing NO and oxidative stress) from the parasite, and (4) NF-kB activation in brain endothelial cells, neurons, and glial cells resulting in caspase 3 activation [8].

Microparticles are cellular membrane-derived vesicles generated by cytoskeletal alterations as a result of cellular membrane remodeling and loss of phospholipid asymmetry. Under physiological conditions, microparticles derived from platelets, white blood cells, red blood cells, and endothelial cells are expressed at normal levels. The overexpression of microparticles is associated with cell activation and apoptosis [17]. The *ATP-binding cassette transporter A1* (*ABCA1*) gene is involved in the process of microparticle release. *ABCA1* gene deletion or knockout is associated with protection from CM in mice [18, 19]. Moreover, endothelial microparticles (EMPs) and TNF are increased during coma episodes of CM in Malawian children compared to the uncomplicated malaria group [20]. This evidence highlights an important role of microparticles in the pathogenesis of CM [17].

The roles of CD8+ T cell in malaria infection are highlighted in many studies. CD8+ T cell acts as (1) effector cell in hepatic and erythrocytic phase for malaria vaccine development [21] and (2) pathogenic cell in experimental cerebral malaria (ECM) development [22]. In CM, it was found that dendritic cells (CD8α+ DCs) are responsible for malaria antigen presentation to CD8+ T cell. This leads to CD8+ T cell proliferation and cytotoxic T lymphocyte (CTL) production mediating ECM. The critical role of CD8α+ DCs as the major antigen-presenting subset in CM was further demonstrated in the protection against CM in CD8α+ DC-depleted mice [21]. The role of CD8+ T cell has been identified as the major mediator of lethality in ECM via the secretion of perforin and granzymes inducing brain endothelial apoptosis and disruption of BBB. In addition, the cytoadherence of pRBCs to brain endothelial cells could lead to malaria antigen uptake via a trogocytosis-like process [22]. Interestingly, brain endothelial cells of PbA-infected mice produced a large number of MP which finally resulted in CM pathogenesis [20].

## Insight into gene regulation of malaria infection

Gene regulation is a process responsible for controlling the rate and manner of gene expression. In humans, dysfunctional gene regulation has been demonstrated in various pathological processes such as inflammatory responses and metabolic processes. Researchers have exerted extensive efforts to uncover the genetic regulation of infectious diseases to develop novel therapeutic agents that target the critical components, which are essential for the survival or amplification of the infective agents or which directly regulate the host immune system. Human gene expression is mainly regulated at the transcriptional and translational levels. Moreover, the mechanisms of epigenetic regulation of gene expression such as chromatin remodeling, histone modification, and non-coding RNAs, particularly microRNAs (miRNAs), have been shown to play important roles in the pathogenesis of complex diseases [20].

miRNAs are 21–25 nucleotide single-stranded non-coding RNA molecules. miRNAs are transcribed from miRNA genes by RNA polymerase II into long pri-miRNAs and then cleaved by the enzyme Drosha into smaller pre-miRNAs. This precursor is subsequently exported to the cytoplasm by the exportin-5/Ran-GTP complex and then further processed by Dicer into double-stranded RNAs. One of the strands is degraded, and the mature miRNA is incorporated with RISC (RNA-induced silencing complex). miRNAs regulate gene expression through complementary binding to the 3′ untranslated region (3′ UTR) of the targeted messenger RNA (mRNA). If the complementary paring pattern between the miRNA and the targeted mRNA is perfect, then the mRNA is cleaved by RISC, whereas if the paring is imperfect, then translational repression occurs [23].

A role for miRNAs in malaria pathogenesis has been increasingly documented. Transcriptome-wide analysis of miRNAs in *Anopheles gambiae* showed the differential expression levels between blood meal and after infection by *P. berghei* [24]. In sickle cell anemia, miRNAs from sickle cells translocated into *P. falciparum* and inhibited parasite growth [25]. The study of miRNA expression in ECM and non-ECM revealed that miRNAs, e.g., let7i, miR-27a, and miR-150, are significantly differentially expressed in the brains of PbA-infected CBA mice [26]. miRNAs have been reported to control the expression of genes related to malaria pathogenesis in diverse diseases such as the inhibition of ICAM-1 by miR-17-3p, VCAM-1 by miR-126, and E-selection by miR-31 [27]. The apoptosis of endothelial cells, neuroglial cells, and erythroid cells is regulated by miR-29b, the miR-15-16 cluster, the let-7/miR-98 family, and the miR-17-92 cluster [28]. NF-kB signaling is inhibited by miR-181b and miR-146a [29–31]. Hypoxia is regulated by miR-210 [29]. In addition, miRNA array analysis of postmortem kidney samples from malaria patients with acute kidney injury has also been investigated [32]. Therefore, this evidence highlights the potential role of miRNAs in the pathogenesis of malaria. However, the study of miRNA in human CM is still limited. Ultimately, the application of miRNA as inhibitor of pathogenic gene expression should be further studied as a new approach for adjunct CM therapy.

## Treatment and prevention of CM

The early diagnosis and urgent treatment of complicated malaria is a good practice for the prevention of CM. The complicated malaria case must be considered as a medical emergency and high priority of treatment. The recommended antimalarial treatment for complicated malaria including CM is water-soluble artemisinin derivative artesunate. The supportive management of malaria complication is essential such as fluid and electrolyte imbalance and convulsion. Many clinical trials studied on adjunctive therapies for complicated malaria using exchange blood transfusion, anti-TNF, etc. However, no effective adjunctive therapies demonstrated the improvement of severity outcome [33].

The control programs of malaria are environmental insecticide-spraying programs, insecticide-treated bed nets, drug treatment in the case of clinical infection, and prophylactic measure for travelers. However, the major problems of the control programs include multidrug resistance in parasites, insecticide (pyrethroids) resistance in mosquitos, and the lack of effective vaccines. *P. falciparum* resistance to multiple drugs has been widely detected in endemic areas. The failure of artemisinin treatment occurred in five countries of the Greater Mekong sub-region: Cambodia, Myanmar, Thailand, Vietnam, and the Lao People’s Democratic Republic [2]. Drug resistance in the malaria parasite is due to mutations of genes that encode the critical components of the drug target such as *P. falciparum* dihydrofolate reductase (Pfdhfr) [34–38].

The current approach for malaria vaccine development is based on the use of recombinant proteins or the attenuated whole organism. However, there is no practical or effective vaccine in clinical use. The key considerations are the delivery system, evolutionary changes in the parasites, and the development of resistance. After vaccination, humans have two main responses of antiparasite and antitoxic immunity. Vaccines are designed for various stages of the malaria life cycle: the pre-erythrocytic-stage vaccines for the prevention of infection, the blood-stage vaccines for the prevention of clinical illness and death, and the sexual stage vaccines for blocking transmission. The most advanced candidate vaccine for *P. falciparum* (RTS, S-AS01) is in phase III efficacy trials [39].

## Human genetics of CM

The risk factors of CM are age, immunological status, overall health status of the host, variation in host genotype, and parasite diversity [40]. The specific population risk groups are young children in stable transmission areas, non-immune pregnant women, semi-immune pregnant women in areas of high transmission, people with HIV/AIDS, and international travelers from non-endemic areas [3]. Human genetic factors influence the outcome of malaria severity. CM predisposition could be explained by genetic polymorphisms of the genes involved in malaria pathobiology. Most of the malaria-related genes involve immunological responses and cell receptors. Single nucleotide polymorphisms (SNPs) in the promoter region of the *TNF-α* gene are significantly associated with susceptibility to CM in the Gambia, Africa, Myanmar, and Thailand [41, 42]. However, variation in the genetic background of each ethnic population contributes to the differences in the level of disease predisposition and severity. Candidate gene-based genetic association studies have been extensively analyzed in various populations (Table 3).

**Table 3:** Genetic variants associated with CM in Asians

No.	Gene	SNP/region	*P* value	Odds ratio	Population	Ref.
1	*ABCA1*	−477 and −320/promoter	<0.0001	5.5	Indian	[51]
2	*CR1*	intron 27, exon 22	0.0003	3.03	Indian	[52]
		rs9429942/promoter	0.0009	0.25	Thai	[53]
3	*TNF-α*	−308	0.04	ND	Gambia	[54]
		−238, −308, −857, −863, and −1031/promoter haplotype	<0.001	34.5	Myanmar	[55]
4	*TIM1*	rs7702919, rs41297577, rs41297579/promoter haplotype	0.0009	0.41	Thai	[56]
5	*ICAM-1*	K29M/N-terminal domain	>0.05	ND	Thai	[57]
		K29M/N-terminal domain	0.0042	1.39	Kenyan	[58]
6	*HO-1*	Promoter	>0.05		Thai, Karen Myanmar	[59]
		Promoter	<0.008	3.14	Myanmar	[60]
7	*PECAM-1*	125 V/V and 563 N/N/coding region	<0.01	2.92	Thai	[61]
8	*CD36*	539delAC	0.04	ND	Thai	[62]
		−14/promoter	0.016	0.62	Thai	[63]
		−53/promoter	0.05	0.68	Thai	[63]

The human genetic variations/phenotypes associated with malaria infection are mainly demonstrated in the host red blood cells, and the immunological components include (1) red blood cell/hemoglobin defects such as thalassemia, sickle cell trait, HbC, and HbE; (2) enzyme defects such as G6PD deficiency and PK deficiency; (3) membrane defects such as ovalocytosis; (4) the Duffy blood group; (5) immunogenetic variants such as HLA alleles; and (6) immunological components such as complement receptor 1, NOS2, TNF-α, and the chromosome 5q31–q33 region (the cytokine region). These genetic variants are highly detected in malaria-endemic areas as a result of natural selection. The mechanism of protection is not yet clear. The reduction or failure of parasite invasion/multiplication in the red blood cells, induction of clearance by the immune system, and increased oxidative stress are responsible for malaria protection [43, 44].

## Parasite genetics of CM

Parasite genetics may also be involved in CM because *P. falciparum* transforms host red cell membranes by incorporation of parasite-derived proteins with the erythrocyte cytoskeleton. The key virulence proteins are *P. falciparum* erythrocyte membrane protein 1 (PfEMP1) and knob-associated histidine-rich protein (KAHRP), which involve the formation of a knob structure linked to the red blood cell cytoskeleton. The development of PfEMP1 initiates from the trophozoite stage, and the knob structure that is formed adheres to the endothelial cell of the host [45, 46]. The parasite gene called “*var*” is extremely diverse and encodes PfEMP1 that binds to various receptors on the host. The *var* gene contains two exons encoding Duffy binding-like (DBL), cysteine-rich interdomain region (CIDR), N-terminal sequence (NTS), and acidic terminal sequence (ATS) domains [47, 48]. The different domains of PfEMP1 contribute to the variety of antigenic adhesion molecules and are responsible for different clinical consequences (Table 4). The parasite evades the host immune system by intergenic recombination to generate genetic variants. A substantial level of polymorphisms was observed in *Csp* (circumsporozoite protein) and Msp-1 and Msp-2, which encode the merozoite surface proteins 1 and 2, respectively [49, 50]. Moreover, mutations in genes related to treatment failure enhance the risk of CM.

**Table 4:** Parasite ligands, host receptors, and clinical consequences [61–63]

Receptor	PfEMP1 domain	Clinically significant
CD36	CIDR1α	Uncomplicated/severe malaria
ICAM-1	DBL2/β type	Cerebral malaria
CSA	DBL3/γ type, CIDR1α	Placental infection and other forms
HS	DBL1α	RBC rosette formation
CR1	DBL1α	Rosette/immune complex formation/parasite invasion
CD31/PECAM	CIDR1α, DBL2/δ type	Virulence-associated endothelial receptor

## Conclusions

CM is a fatal multifactorial disease, and approximately 20 % of patients will die from coma and seizure if no effective treatment is available. Multidrug resistance is still a problem in endemic areas, and effective vaccines are not fully developed. Now, geneticists are exerting extensive efforts to investigate the causative genes/markers of CM. Because more than 60 % of human protein-coding genes are predicted to be regulated by miRNAs, miRNAs may be involved in the mechanism of CM. The utilization of quantitative and qualitative analyses of miRNAs in CM will facilitate the discovery of new interventions for diagnostic and therapeutic purposes.

## Abbreviations

ATS: Acidic terminal sequence
CIDR: Cysteine-rich interdomain region
CM: Cerebral malaria
*Csp*: Circumsporozoite protein
DBL: Duffy binding-like
GPI: Glycosylphosphatidylinositol
ICAM-1: Intercellular adhesion molecule-1
IFN-γ: Interferon-γ
KAHRP: Knob-associated histidine-rich protein
memTNF: Transmembrane form of TNF
miRNAs: MicroRNAs
Msp-1: Merozoite surface protein 1
Msp-2: Merozoite surface protein 2
NTS: N-terminal sequence
PbA: *P. berghei* ANKA
PfEMP1: *P. falciparum* erythrocyte membrane protein 1
pRBCs: Parasitized red blood cells
SNPs: Single nucleotide polymorphisms
TNFR2: Tissue necrotic factor receptor 2
TNF-α: Tumor necrosis factor alpha
